# Development of a non-invasive fibrosis test for chronic hepatitis B patients and comparison with other unpatented scores

**DOI:** 10.1186/1756-0500-6-212

**Published:** 2013-05-27

**Authors:** Chao-Wei Hsu, Kung-Hao Liang, Shiu-Feng Huang, Kuo-Chien Tsao, Chau-Ting Yeh

**Affiliations:** 1 Liver Research Center, Chang Gung Memorial Hospital, 199, Tung Hwa North Road, Taipei, Taiwan; 2 Department of Pathology, Chang-Gung Memorial Hospital, Taoyuan, Taiwan; 3 Department of Medical Biotechnology and Laboratory Science, Research Center for Emerging Viral Infections, Chang Gung University, Taoyuan, Taiwan; 4 Departments of Laboratory Medicine, Chang Gung Memorial Hospital, Taoyuan, Taiwan

**Keywords:** Hepatitis B, Fibrosis, Score, Receiver operation characteristic curve

## Abstract

**Background:**

Despite the availability of patented non-invasive methods, evaluation of the degrees of liver fibrosis remains difficult when conducting a retrospective study. Such inadequacy is largely caused by requirement of biochemical parameters rarely performed in routine clinical tests. We developed a novel fibrosis HB-F score using commonly performed tests for HBV infected patients.

**Methods:**

424 patients with chronic HBV infection were included. Using clinical and virological data, HB-F score was developed from a training cohort (n = 213) and validated in a separate cohort (n = 211). The performance was compared with five other unpatented scores using ROC curves.

**Results:**

Univariate and multivariate analysis revealed that age, AST/ALT ratio, platelet count and prothrombin time prolongation were significantly associated with the ISHAK fibrosis score, and were used to calculate the HB-F score. When HB-F was used to assess prominent fibrosis and cirrhosis, the AUC was 0.81 and 0.80 respectively in the training cohort, and 0.80 and 0.76 respectively in the validation cohort. HB-F had the highest AUC compared with other scores. Furthermore, in assessing paired liver biopsies with increase or decrease of ISHAK scores, HB-F showed significant change in the same direction.

**Conclusions:**

A new non-invasive score was developed, which could be used to assess severity of liver fibrosis in retrospective longitudinal studies in HBV patients.

## Background

It has been estimated that 350 millions patients are infected by hepatitis B virus (HBV) worldwide [1]. Chronic HBV infection may lead to severe sequelae such as liver fibrosis and cirrhosis [1]. Liver fibrosis is a progressive damage which not only impairs liver functions but also increases the risk of hepatocellular carcinoma [2]. The progression of fibrosis has multiple stages which are commonly defined by ISHAK or METAVIR scoring systems [3-5]. An advanced fibrosis score represents a serious clinical condition which requires careful medical managements. To prevent disease progression, we need to detect liver fibrosis at an earlier stage and to provide adequate antiviral agent treatment [6]. In the past decades, liver biopsy has been a gold standard for the assessment of fibrosis stages. However, there are several limitations when applying this method, such as sampling bias, low platelet counts, prolonged prothrombin time, massive ascites, patient’s intention and compliance as well as scoring variations from different pathologists [7]. Thus, other methods to accurately assess liver fibrosis are continuously sought, especially for non-invasive tests.

Ultrasound is one possible alternative for non-invasive assessment. FibroScan and Acoustic radiation force impulse (ARFI) elastography have been used for evaluation of fibrosis in chronic hepatitis B or C patients. However, it was found that the accuracy of FibroScan and ARFI was greatly interfered by higher degrees of necroinflammation and more advanced stages of fibrosis or cirrhosis especially in chronic hepatitis B patients [8-11].

Recently, a number of fibrosis scores have been proposed as surrogates to liver biopsy. These scores are often composed of a combination of biochemistry measurements and clinical parameters. For example, the AAR score is based on the ratio of aspartate transaminase (AST) and alanine transaminase (ALT) values [12]. The APRI score is the AST value divided by platelet counts [13]. The early AAR and APRI scores motivate several subsequent scores where the ratios of AST/ALT or AST/platelet counts are employed as part of their equations. The Fibrosis index (FI) score is composed of platelet counts and serum albumin [14]. The Fibroindex consists of AST, platelets, and gamma globulin measurements [15]. The FIB-4 index includes age, AST, ALT and platelet counts [16,17]. A patented test, named FibroTest, is composed of alpha2-macroglobulin, haptoglobin, gamma glutamyl transpeptidase (GGT), age, bilirubin, apoA1, and sex. Most of these markers were initially derived from patients with chronic hepatitis C but were subsequently tested in chronic hepatitis B patients. Meta-analysis for the performance of biomarkers in HBV or hepatitis C virus (HCV) infection showed that assessment of the treatment efficacy on fibrosis progression was equally effective when estimated by either FibroTest or biopsy. Despite the great performance of FibroTest, there were still no sufficient data to show that biomarker or biopsy alone could make accurate fibrosis staging in patients with chronic HBV infection [18]. Furthermore, the requirement of measuring several uncommon tests to calculate the score greatly limited its use in retrospective study [19-21]. We were thus motivated to formulate a new hepatitis B-fibrosis score (HB-F) using commonly performed tests and clinical data in a large cohort of chronic HBV infected patients.

## Methods

### Patients and samples

Under approval of Institutional Review Board, Chang Gung Medical Council, this study was conducted at Liver Research Center, Chang Gung Memorial Hospital, Taiwan. A total of 424 adult patients with compensated chronic hepatitis B were recruited (Table 1). All of them have signed informed consent forms. They received liver biopsy between January 2007 and July 2009. No antiviral agents were given to these patients before biopsy was taken and patients with other viral co-infections, such as HIV, hepatitis C or hepatitis D co-infections were pre-excluded. Hepatitis B e Antigen (HBeAg) and antibodies to HBeAg (anti-HBe) were measured before liver biopsy. Biochemistry were assessed at the same time of liver biopsy, including AST, ALT, bilirubin, gamma glutamyl transpeptidase (GGT), albumin, alpha-1 globulin, alpha-2 globulin, beta globulin, gamma globulin and albumin/globulin ratio. Hematology tests were also performed at same time point, including hemoglobin, platelet count, white blood cell, prothrombin time, and alpha-fetoprotein (AFP).

**Table 1:** Basic clinicopathological data for hepatitis B patients included

**Characteristics**	**Training cohort**	**Verification**
	**(n** = **213)**	**cohort (n** = **211)**
Age at biopsy in years, mean ± SD	44.4 ± 11.8	45.6 ± 11.4
Male, n (%)	179 (84)	175 (83)
Biopsy		
Necroinflammation score, mean ± SD		
Periportal inflammation	1.0 ± 0.8	0.9 ± 0.8
Confluence necrosis	0.3 ± 1.0	0.2 ± 0.8
Focal inflammation	1.9 ± 0.7	2.0 ± 0.7
Portal inflammation	2.2 ± 1.0	2.2 ± 0.9
ISHAK fibrosis score, n (%)		
0	1 (1)	1 (1)
1	28 (13)	28 (13)
2	32 (15)	32 (15)
3	70 (33)	71 (34)
4	21 (10)	21 (10)
5	46 (22)	45 (21)
6	15 (7)	13 (6)
HBeAg-positive, n (%)	109 (51)	91 (43)
Biochemistry, mean ± SD		
AST (IU/mL)	91.3 ± 83.6	94.9 ± 100.1
ALT (IU/mL)	170.1 ± 173.8	166.2 ± 177.9
AST/ALT	0.6 ± 0.3	0.6 ± 0.3
AFP (ng/mL)	10.5 ± 22.2	20.4 ± 82.0
Bilirubin (mg/dL)	1.0 ± 0.7	1.0 ± 0.4
GGT (IU/L)	69.0 ± 77.0	60.1 ± 57.3
Albumin (g/dL)	4.6 ± 0.3	4.5 ± 0.4
Platelet (×10^3^/mm^3^)	188.2 ± 54.2	189.2 ± 49.4
Prothrombin time prolongation (sec)	1.0 ± 0.6	1.0 ± 0.8
White blood cell (×10^3^/mm^3^)	5.7 ± 1.7	5.5 ± 1.6
Hemoglobin (g/dL)	15.1 ± 1.4	15.2 ± 1.3
Alpha-1 globulin (g/dL)	0.2 ± 0.1	0.2 ± 0.0
Alpha-2 globulin (g/dL)	0.8 ± 0.2	0.8 ± 0.1
Beta globulin (g/dL)	0.8 ± 0.1	0.7 ± 0.1
Gamma globulin (g/dL)	1.5 ± 0.4	1.4 ± 0.4
Albumin/Globulin	1.5 ± 0.3	1.5 ± 0.3

Histologic evaluation of biopsy samples were carried out at the Pathology Department, according to ISHAK’s staging system, which had seven severity levels: FS0 (no fibrosis), FS1 (fibrous expansion of some portal areas), FS2 (fibrous expansion of most portal areas), FS3 (fibrous expansion of most portal areas with occasional portal to portal bridging), FS4 (fibrous expansion of most portal areas with marked bridging), FS5 (marked bridging with occasional nodules, incomplete cirrhosis), and FS6 (cirrhosis).

After biopsy, subjects of each ISHAK stage were randomly assigned into two cohorts: the training cohort (n = 213) and the validation cohort (n = 211), for formula-deriving and validation purposes respectively. This way, subjects were evenly split across all ISHAK stages. Clinical parameters and biochemistry measurements were individually tested for their association to the ISHAK fibrosis stages. The associated factors were then combined to produce the HB-F score. The score was then validated using the validation cohort, and its performance was compared with five other reported scores (AAR, FIB-4, FI, APRI, Fibroindex), which were all originally derived from hepatitis C infected patients.

### Statistical analysis

Clinicopathological data distribution was compared between the training and validation cohorts by either Chi-square tests or two-sample t-tests with unequal variance. Linear regression was used for the univariate and multivariate analysis to assess the association between the clinical/ laboratory parameters and ISHAK fibrosis scores. Significance levels of correlation were assessed by Wald test statistics. All the P-values were two-tailed. The Receiver Operating Characteristic (ROC) Curve was used to examine the trade-off of sensitivity and specificity. The performance of classification was assessed by the Area Under Curve (AUC). The HB-F and FIB-4 scores of the two biopsy examinations were assessed by paired t-tests.

## Results

The distributions of subjects’ clinicopathological values were presented in Table 1. The two cohorts had similar number of subjects across all ISHAK stages without significant disparity (P = 1.000). Additionally, comparison of the age, gender, inflammation scores and the biochemistry and hemogram values revealed no significant difference.

Univariate and multivariate associations of various factors to ISHAK fibrosis stages were performed in the training cohort (Table 2). Significant associations were found in eight parameters, including age at biopsy, HBeAg, AST/ALT ratio, AFP, GGT, Albumin, platelet and prothrombin time prolongation (P < 0.05). Among them, four parameters (age at biopsy, AST/ALT ratio, platelet and prothrombin time prolongation) showed stronger association (P < 0.001). A multivariate analysis of the four parameters showed that all of them remained significant (P < 0.05), indicating their independent association to the ISHAK fibrosis stages.

**Table 2:** Univariate and multivariate analysis for factors associated with liver fibrosis

**Characteristics**	**Beta (95% CI)**	**P**	**Adjusted beta (95% CI)**	**P**
Age at biopsy	0.035 (0.018, 0.052)	< 0.001	0.018 (0.001, 0.036)	0.044
Male	0.046 (-0.597, 0.506)	0.870		
HBeAg-positive	-0.496 (-0.943, -0.048)	0.030		
AST (IU/mL)	0.001 (-0.001, 0.004)	0.363		
ALT (IU/mL)	0.000 (-0.001, 0.001)	0.802		
AST/ALT	1.881 (1.199, 2.563)	< 0.001	1.085 (0.368, 1.803)	0.003
AFP (ng/mL)	0.012 (0.001, 0.023)	0.026		
Bilirubin (mg/dL)	0.142 (-0.168, 0.452)	0.367		
GGT (IU/L)	0.006 (0.002, 0.009)	0.003		
Albumin (g/dL)	-1.440 (-2.839, -0.042)	0.044		
Platelet (×10^3^/mm^3^)	-0.012 (-0.015, -0.009)	< 0.001	-0.009 (-0.013, -0.005)	< 0.001
Prothrombin time prolongation (sec)	0.781 (0.404, 1.157)	< 0.001	0.449 (0.098, 0.800)	0.012
White blood cell (×10^3^/mm^3^)	0.049 (-0.093, 0.192)	0.495		
Hemoglobin (g/dL)	0.053 (-0.125, 0.231)	0.558		
Alpha-1 globulin (g/dL)	-0.898 (-2.683, 0.886)	0.322		
Alpha-2 globulin (g/dL)	-0.505 (-1.746, 0.736)	0.423		
Beta globulin (g/dL)	-0.808 (-2.160, 0.545)	0.240		
Gamma globulin (g/dL)	0.214 (-0.327, 0.754)	0.436		
Albumin/Globulin	-0.422 (-1.180, 0.335)	0.273		

The four parameters were then linearly combined to generate the HB-F score using the multiple regression coefficients (Adjusted Beta) in Table 2, omitting the constant term. The formula of HB-F score was written as:

HB-F = 0.018 × [Age] + 1.085 × [AST/ALT] - 0.009 × [Platelet (×10^3^/mm^3^)] + 0.449 × [Prothrombin time prolongation (sec)]

**Figure 1:**
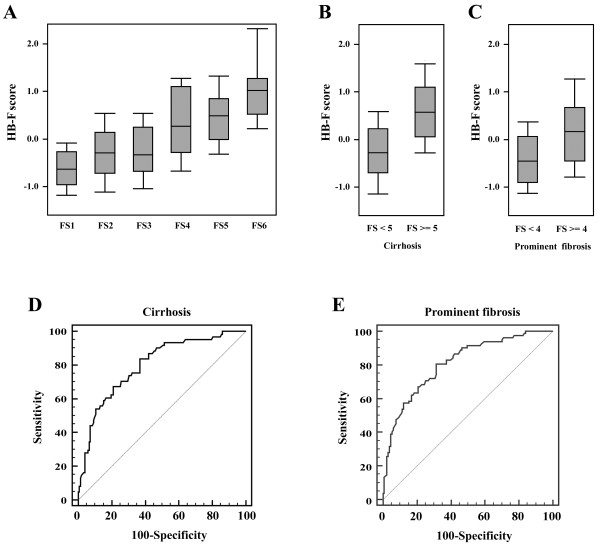
**Performance of the HB**-**F fibrosis score in the training cohort of patients.** (**A**) The ranges of HB-F scores in patients with different ISHAK fibrosis score (FS1-FS6). Gray boxes represented the 25%-75% quartiles; The middle horizontal lines represented the median scores. (**B**) The ranges of HB-F scores in subjects stratified by FS < 5 (non-cirrhotic patients) and FS > =5 (cirrhosis) (P < 0.001). (**C**) The ranges of HB-F scores in subjects grouped by FS < 4 and FS > =4 (prominent fibrosis) (P < 0.001). (**D**) The ROC curve of the HB-F score for the diagnosis of cirrhosis patients (AUC = 0.799). (**E**) The ROC curve for the diagnosis of prominent fibrosis patients (AUC = 0.810).

In the training cohort, subjects with a more severe fibrosis stage also had a higher range of HB-F score (Figure 1). The score was then used to classify patients with (FS > =5) or without cirrhosis (FS <5) and patients with (FS > =4) or without severe fibrosis (FS < 4). The AUCs were 0.810 and 0.799 for the distinction of severe fibrosis and cirrhosis, respectively, in the training cohort. Consistently, the corresponding AUCs were 0.797 and 0.757 respectively in the verification cohort. Both AUCs were larger than 0.75 suggesting adequate capability of HB-F in identifying severe fibrosis subjects (Figure 1).

**Figure 2:**
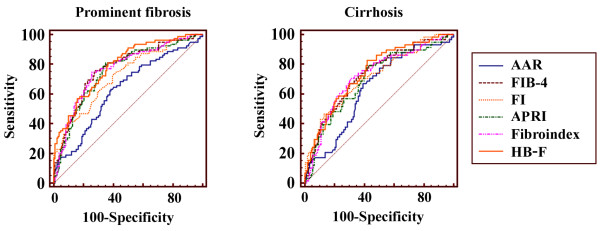
**The ROC curves of HB-F score and 5 other methods (AAR, FIB-4, FI, APRI, and Fibroindex) for the diagnosis of prominent fibrosis (left) or cirrhosis (right) in the validation cohort.**

When compared with five other reported methods, HB-F had the highest AUC (Table 3, Figure 2). The next three best scoring methods were FIB-4, Fibroindex and APRI, respectively, but all had no statistically significant difference when compared with HB-F score. AAR, on the other hand, had a significantly inferior performance in classifying both the cirrhosis and prominent fibrosis. FI had an inferior performance in identifying prominent fibrosis. The ROCs of all the methods were presented in Figure 2.

**Table 3:** Performance of six fibrosis score for evaluation of liver fibrosis and cirrhosis in patients with chronic hepatitis B

**Non-invasive index**	**AUC (95% CI)**	Difference between areas (95% CI)*	P*
Prominent fibrosis			
HB-F (training)	0.810 (0.751, 0.861)		
HB-F (verification)	0.797 (0.737, 0.849)		
FIB-4	0.772 (0.709, 0.826)	0.0255 (-0.0236, 0.0746)	0.3087
Fibroindex	0.767 (0.704, 0.822)	0.0301 (-0.0298, 0.0899)	0.3251
ARPI	0.753 (0.689, 0.810)	0.0439 (-0.0302, 0.1180)	0.2454
AAR	0.630 (0.561, 0.696)	0.1670 (0.0947, 0.2390)	< 0.0001
FI	0.724 (0.659, 0.784)	0.0728 (0.0093, 0.1360)	0.0246
Cirrhosis			
HB-F (training)	0.799 (0.739, 0.851)		
HB-F (verification)	0.757 (0.694, 0813)		
FIB-4	0.727 (0.662, 0.786)	0.0297 (-0.0223, 0.0818)	0.2624
Fibroindex	0.722 (0.656, 0.781)	0.0351 (-0.0320, 0.1020)	0.3054
ARPI	0.698 (0.631, 0.759)	0.0589 (-0.0244, 0.1400)	0.1556
AAR	0.636 (0.568, 0.701)	0.1210 (0.0470, 0.1950)	0.0013
FI	0.717 (0.651, 0.777)	0.0403 (-0.0283, 0.1090)	0.2497

*Comparison between the *AUC* of *HB*-*F* (verification cohort) and those of the other scoring methods.

**Figure 3:**
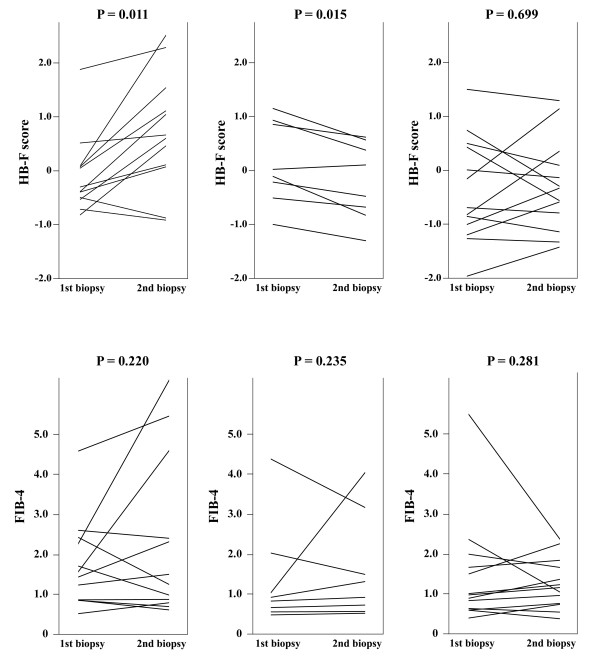
**Comparison of HB-F and FIB-4 scores in determination of improvement or deterioration of liver fibrosis in paired biopsies.** Left, The second biopsy showed an increase of ISHAK fibrosis level (n = 12). Middle, The second biopsy showed an decrease of ISHAK fibrosis level (n = 8). Right, The second biopsy showed the same ISHAK fibrosis level (n = 13).

A total of 33 subjects had received a second biopsy and biochemistry examinations. They offered additional disease progression and remission data for evaluating the change of HB-F and FIB-4 score under an increase or decrease of ISHAK levels (Figure 3). FIB-4 was chosen because it had the highest AUC among the benchmark scores. The HB-F scores showed significant changes in the same direction of an increment (P = 0.011) and decrement (P = 0.015) of ISHAK levels. In contrast, FIB-4 did not show such a correlated change.

## Discussion

In this study, a novel fibrosis score named HB-F was formulated, by combination of four factors which was highly significant in the association with ISHAK fibrosis stages (P < 0.001). The HB-F score was developed from a large cohort of HBV-infected patients, as HBV infection is a major etiology of fibrosis in Taiwan. Although the study also included other HBV-related factors such as the presence or absence of HBeAg, this factor only manifested marginal association to the ISHAK stages (P = 0.03). Hence, the HBV-related factors were not included in HB-F.

The four factors in HB-F score included age, the AST/ALT ratio from biochemistry test, platelet count and prothrombin time prolongation from hematological test. The prothrombin time prolongation was the only factor not previously included in other scores. Our multivarite analysis suggested that it was an independently associated factor to liver fibrosis. One limitation of this study is that the performance is only assessed in HBV-related patients. This is due to the high prevalence of HBV infection in Taiwan and thus a need to develop reliable fibrosis score for retrospective studies. Nevertheless, the four factors in the equation are not HBV-specific. Therefore, this score may be used to evaluate the fibrosis of other etiologies such as HCV infection and alcohol, once the corresponding validation is completed in these subjects.

The proposed HB-F score was constructed by multiple regression coefficients (Adjusted Beta). It is a challenge for both pathologists and fibrosis scores alike to discern the stage of early fibrosis (FS1 to FS3). This is also a limitation of HB-F. However, Figure 1A showed the distribution of HB-F score increased as the ISHAK-stage ascended from FS1 to FS2. This observation suggested that HB-F might potentially be used for classifying the very earlier stage fibrosis. A larger number of patients in these two stages are needed for verification.

The HB-F score had superior performance than five other HCV-derived scores in identifying both the prominent fibrosis and cirrhosis patients, when assessed by AUCs. However, FIB-4, Fibroindex and APRI all showed very similar performance as in Figure 2 and Table 3, particularly FIB-4 seemed to be almost equally effective. However, when the scores were evaluated using paired liver biopsies, HB-F proved to be a better score for judgment of histology improvement or deterioration. Additionally, a similar performance between HB-F and other previous scores (FIB-4, FI, Fibroindex) suggested that we could use these scores interchangeably for estimation of fibrosis severity in retrospective studies where several clinical parameters were missing for one of these scores. In other words, our studies and the novel score provided flexibility for retrospective studies where fibrosis evaluation was often limited by data availability.

## Conclusions

In conclusion, we have formulated a new fibrosis score, the HB-F, for chronic hepatitis B patients, by use of a linear combination of age, AST/ALT ratio, platelet count and prothrombin time prolongation values. Judging from the AUC comparison, HB-F score is better than other existing unpatented scores derived from hepatitis C patients.

## Abbreviations

AFP: Alpha-fetoprotein; ALT: Alanine transaminase; AST: Aspartate transaminase; AUC: Area under the curve; FI: Fibrosis index; GGT: Gamma glutamyl transpeptidase; HB-F: Hepatitis B-fibrosis; HBV: Hepatitis B virus; HCV: Hepatitis C virus; ROC: Receiver operation characteristic.

## Competing interests

We declare that we have no competing interests.

## Authors contributions

CWH, designed and conducted the project, analyzed and interpreted the data, drafted the manuscript. KHL, analyzed and interpreted the data, statistical analysis. SFH, handled tissue samples and interpreted the pathological data. KCT, handled blood samples and interpreted the serological data. CTY, medical oversight of the whole study implementation, co-designed and supervised the conduct of the project, co-drafted and revised the manuscript. All authors read and approved the final manuscript.
